# Artificial Intelligence and Publishing Ethics: A Narrative Review and SWOT Analysis

**DOI:** 10.7759/cureus.84098

**Published:** 2025-05-14

**Authors:** Pooja Gurnal, Lokesh Rana

**Affiliations:** 1 Anesthesia, All India Institute of Medical Sciences, Bilaspur, Bilaspur, IND; 2 Radiology, All India Institute of Medical Sciences, Bilaspur, Bilaspur, IND

**Keywords:** artificial intelligence, chatbots, large language models (llms), publishing ethics, swot analysis

## Abstract

Artificial intelligence (AI), after surviving two major AI winters (1974-1980 and 1987-2000), is now growing at an exponential rate. This rapid advancement, particularly in its application to medical science and literature, has significantly transformed how research is conducted. The large language tools can produce highly realistic text, enabling diverse tasks with broad applications. In other words, their responses resemble human answers to human questions; however, their malicious use poses serious challenges to scientific research integrity and literature, especially when outputs influence human life, the ethical compass gains more importance than the benefits.

This review aims to provide a comprehensive narrative review of AI, in particular the emergence of large language models and their impact on healthcare scientific research, with a focus on the challenges it poses to ethics and scientific integrity. In addition, it aims to discuss the evolving guidelines from various international organizations on authorship, transparency, and the responsible use of AI.

Databases such as PubMed, Cochrane, Scopus, and Google Scholar were searched to provide a comprehensive review from the published literature on the emergence of AI in the healthcare research setting, along with its positive and negative impacts on research ethics. We also performed a SWOT (Strengths, Weaknesses, Opportunities, and Threats) analysis of AI in research publications and evaluated the ethical challenges it poses.

Chatbots are AI-based conversational large language models, which are proving to be of significant importance in healthcare education, practice, and research. However, caution needs to be exercised in its malicious fabricated use. Organizations such as the Committee on Publication Ethics, World Association of Medical Editors, Journal of the American Medical Association, and International Committee of Medical Journal Editors state that chatbots do not qualify as co-authors, with only responsible and ethical use of AI being permitted. Caution needs to be exercised at the individual level by academics when they use these tools, and they should be transparent in their disclosure of their use. The advent of Google revolutionized scientific research, and similarly, AI-assisted chatbots represent the next leap forward. Hence, it is crucial to use these tools with caution, accountability, and transparency. Through this narrative review, we aim to guide researchers in understanding new guidelines and approaches to research ethics in this fast-evolving era of AI.

## Introduction and background

Artificial intelligence (AI), a field of computer science and linguistics, aims to develop machines capable of performing tasks that typically require human intelligence, thus smartly incorporating human behavior and intelligence into machine systems [1,2]. AI has evolved into a broad transdisciplinary field with transformative influence in fields concerned with logic, statistics, cognition, neuroscience, cybernetics, and computer engineering [3].

The origin of AI can be traced back to 1950, when British mathematician Alan Turing published his work titled “Computing Machinery and Intelligence,” where he proposed the question, “can machine think?” and introduced the concept now known as the Turing Test, a method to evaluate a machine’s ability to exhibit intelligent and critical behavior simulating the human brain [3]. Building upon Turing’s foundational ideas, the field of AI was formally established in 1956 during the Dartmouth Summer Research Project on Artificial Intelligence. This pivotal workshop, organized by McCarthy and other prominent researchers, marked the first use of the term “Artificial Intelligence.” McCarthy defined AI as “The science and engineering of making intelligent machines,” thus setting the stage for decades of research into machine learning, reasoning, and problem solving [4]. A significant advancement in AI technology is the development of chatbots powered by large language models (LLMs). These AI-driven programs simulate human-like text or voice interactions, enabling automated communication and task execution. The first chatbot was ELIZA, developed in mid-1960 by Joseph Weizenbaum at the Massachusetts Institute of Technology. ELIZA was a natural language processing (NLP) program that simulated back-and-forth conversation by using pattern matching and substitution rules. After prolonged AI winters in between and sluggish developments in AI technology, the COVID-19 pandemic exposed some critical gaps in existing digital health services. This led to the need for AI-powered LLMs surfaced and accelerated the development of conversational intelligence [5-9]. In November 2022, OpenAI (San Francisco, CA, USA) released ChatGPT, a chatbot with an advanced NLP tool based on the generative pre-trained transformer (GPT) model, which has been trained on a large amount of text data and simulates human understanding and response.

Since their inception, AI chatbots have significantly impacted healthcare education, academic writing, research, and clinical practice [7]. Authors, reviewers, and editors are increasingly leveraging generative AI to enhance various aspects of scientific publication [10-12]. The responsible use of these LLMs offers benefits such as improved writing efficiency, enhanced language quality, and the ability to analyze extensive datasets such as electronic health records [13,14]. Moreover, AI chatbots help overcome language barriers, enabling non-native English-speaking researchers to write, edit, and peer review scientific work more efficiently. Additionally, these tools can assist native English-speaking patients by simplifying complex medical information, thereby improving patient understanding and engagement [15,16]. However, these LLMs are prone to making errors and providing misleading information, especially on topics that require specific technical information, as they may be trained with limited data and may be fluent, but many times not factual [17,18]. The unreliability associated with LLMs is largely because these are trained on statistical patterns of language available online, which can be false, fabricated, malicious, or outdated [18]. Other caveats include responsibility and authorship, as these LLMs are incapable of taking responsibility. Serious concerns include risk of bias, plagiarism, lack of originality, cybersecurity, and infodemic risk. Other ethical risks include data privacy and data governance, which may negatively impact the trust placed on health professionals [8,9]. These needed course corrections, mitigation, and robust guidelines for their use will likely have a detrimental effect on an overtly competitive publishing ecosystem. The current Committee on Publication Ethics (COPE)/International Committee of Medical Journal Editors (ICMJE)/Cochrane guidelines make these chatbots unqualified to be listed as co-authors in research articles [19,20]. According to ICMJE guidelines, for authorship, four criteria need to be fulfilled, which include substantial contribution in conception, design, and acquisition; drafting and critical review of intellectual content; final approval; and accountability for all aspects of the work [21]. All stakeholders in research, i.e., research scholars, editors, journals, and regulating national and international organizations, need to come up with initiatives for standard operating procedures (SOPs) and the code of ethics for the responsible use of these chatbots [20]. This review aims to conduct a critical inquiry about these LLMs, with insights into the ways to overcome the drawbacks of LLMs, along with an overview of the current academic and regulatory guidelines [21].

## Review

Methodology

We searched PubMed, Cochrane, Scopus, and Google Scholar with search terms including “chatbot,” “ChatGPT,” “large language models,” and “AI in scientific publishing” with Boolean operators AND, OR, NOT. English-language articles published in the last five years of publications with full-text availability were considered for inclusion in the review. The inclusion criteria were all peer-reviewed articles, reviews, editorials, and regulatory agency guidelines on AI and LLMs in scientific publishing. The exclusion criteria were non-English-language studies, duplicate records, and conference abstracts. The two reviewers screened the titles and abstracts independently. The full text of potentially relevant studies was reviewed, with disagreements resolved by consensus.

Concerns and challenges

The rise of chatbots powered by LLMs has significantly transformed scientific research and publishing. While these tools offer efficiency and accessibility, their unchecked use raises concerns about the quality and integrity of academic work [21-26].

A fundamental aspect of research practice is integrity, which requires the highest standards of honesty, accuracy, and transparency. AI, despite its advantages, poses concerns, particularly with respect to algorithmic bias, automation ethics, and its implications in research publications. Therefore, AI-based conversational LLMs should be adopted with extreme caution because of their potential limitations [19-21].

Accuracy Issues

The accuracy of chatbots can be challenged. For instance, when ChatGPT was asked to write about the pathogenesis of certain rare metabolic disorders, it documented unreliable and misleading performance [17,27]. This could have been due to a small dataset for training the AI algorithm, as well as the underrepresentation of many geographical regions and countries [18,19].

Factual Errors and Hallucinations

There have been instances when individuals have experimented by asking questions ranging from controversial to specific publishing-related technical questions, where chatbots have provided false and fabricated answers without specific references or with non-existent evidence. Hence, they are fluent but not factual at times [22,23,27-32].

Lack of Transparency

References cited by chatbots are often insufficient and from nonexistent sources. Moreover, as these tools are non-legal entities and their moral responsibility cannot be fixed, they do not qualify for authorship [21,32,33].

Data Falsification and Fabrication

These tools generate a dataset that may look human-generated but can be entirely fictitious. Moreover, these tools are ever-evolving and sophisticated, which makes it difficult to detect errors [23].

Image Manipulation

Misleading image and graph practices, such as replication and deletion, damage the integrity of science as a whole [34,35].

Unrealistic Abstracts

Although these LLMs produce increasingly realistic text, in a study, blinded human reviewers were able to identify generated abstracts in 68% of the cases [36].

Bias

To display the stereotypical gender bias tendency of LLM, when asked to write 10 words associated with men and women in terms of occupation and intelligence, it was quick to display, though with a disclaimer [22,37-42].

All these concerns pose serious threats to the authenticity and credibility of academic work [39,43-45]. An American researcher, Eliezer Y., once said, “by far the greatest danger of AI is that people conclude too early that they understand it.” Hence, this ever-evolving field needs a cautious approach [46]. Erroneous search results can happen if a particular piece of work is not present in the chatbot training set, which can lead to generating irrelevant information [25,46-48]. As LLMs are trained with data that originates largely in economically strong English-speaking countries, the perspectives of other underdeveloped non-English-speaking countries are not being represented [48]. Scholarly benefits of LLMs for non-English-speaking regions have not been adequately addressed in the literature.

Advantages

The exponential advancement of AI has transformed the landscape of healthcare management systems globally, as it has created many opportunities in fields such as genetic research, scientific research, and medical diagnostics. Those involved with scientific publication, be it authors, reviewers,, or editors, can use AI and LLMs in many ways to enhance their work in the scientific publication process [10-12].

Efficiency

AI can augment the efficiency and versatility in writing text, with improved language and analysis of massive data, including electronic health records [13,14].

Language Support

These AI LLMs can overcome the language barriers of researchers from non-native English-speaking regions of the world, who can write and edit their work, as well as reviewers who can peer review others’ work in medical research [15,16].

Data Analysis

LLMs can prove helpful in administrative and documentation tasks involved in research, such as the compilation of medical reports, patient forms, and discharge summaries [24].

Detection of Research Misconduct

Scholarly literature production has its essence in human intelligence and critical thinking. AI tools can improve the quality by the responsible and transparent use of these tools [39]. With the fear of academic dishonesty, AI and LLM models are themselves tools to detect plagiarism, data falsification, and fabrication [40]. Even journals and editors use AI for triaging submissions, validating references, and checking plagiarism and ethical issues [49].

AI and LLMs can be of immense help in the identification of research ideas, writing abstracts and manuscripts, analysis of vast complex data, development of new algorithms for enhancing the human peer review process, and providing researchers a summary of academic literature; however, their use should be mindful and responsible [40]. The complete prohibition of LLMs will hamper diversity and inclusion in research [33].

Regulatory perspective

The widespread use of chatbots and AI tools has led to AI-generated ideas silhouetting seamlessly with original concepts, making it difficult to distinguish between the two [26,35,49]. The lack of transparence as well as accountability has prompted publishing companies to devise guidelines [22,37].

COPE, World Association of Medical Editors (WAME), CSE, and Journal of the American Medical Association (JAMA) network explicitly state in their guidelines that AI tools, such as ChatGPT and other language models, cannot be listed as authors. As AI is not a legal entity, it cannot take responsibility for authorship disputes, claim copyright, or enter into licensing agreements [22,23,25,34].

COPE, ICMJE, and CSE recommend that authors should disclose the use of AI writing tools in the material and method or acknowledgement section [22,23]. Alternatively, authors should mention in the description of the content section the language model tool used, its version, and the name of the manufacturer. However, there is no restriction on the use of basic tools such as checking grammar, spelling, and references [28,29].

These agencies recommend that authors are responsible for the content of the manuscript produced by AI, and there should be adherence to the transparency of the process [23,25]. Companies that publish false research papers are called “paper mills” [11]. COPE has described these as profit-oriented, unofficial, and potentially illegal organizations that produce and sell fraudulent manuscripts that seem to resemble genuine research [12]. The scholarly publishing community has reported its reservations about the misuse of these chatbots in scientific publication [27,29]. The JAMA network journals have also provided guidelines for responsible authorship behavior, with authors to take onus to maintain the integrity of the content generated by AI models and tools for reproduced and recreated material and images [26,28-32,35,49].

Regulatory agencies have devised policies for the use of various analysis software and recommended authors to follow EQUATOR guidelines, and for AI intervention trials, CONSORT-AI and SPIRIT-AI have been recommended [28,31,32]. The Science family of journals has also stated that text, images, figures, or graphics generated by Chatbots are not acceptable [31,32]. LLMs can only be good assistants for experienced researchers who can spot errors in AI-generated information, which, for inexperienced users, can be misleading [18,20,34,37-39]. There must be sound knowledge of the fact that the chatbots provide information that is from a certain timespan and may not be relevant in the present scientific scenario. Further, it can provide nonexistent references, the information provided is not free of plagiarism and bias, and, at present, AI-generated text detectors appear to have a low success rate [31,41]. The developers of these LLMs are expected to explore ways to get certain watermarks into the text generated by AI so that it is distinguishable from human-written text [23,47]. However, regulatory agencies and journals may have limited impact on unethical practices in chatbot-driven publishing, as it is a rapidly evolving area and is largely overlooked in existing reviews and articles. A meta-analysis of 200 government policies and ethical guidelines to determine whether a global consensus exists regarding the ethical principles for AI application inferred 17 common principles in policies and guidelines for AI application [18].

Detection and mitigation

Some prediction tools are available that can differentiate between machine and human-generated text, but the evolving machine learning and LLM tools might overtake them. Chatbot output is detectable as the patterns of words are based on statistical association in the training data, and on careful evaluation, especially when more than a few paragraphs are suggested by it.

Watermarking and Lexical Marking

For the protection of intellectual property, the applications of digital watermarking can prove to be a promising approach. This involves embedding a subtle, structured pattern in word choices or sentence structure using a hidden digital signature within the text metadata. Lexical marking tags involve specific words and phrases subtly inserted in AI-generated content [25].

Cryptography

This encrypts the data, thus increasing its security, transmission, and storage.

Image Integrity Tool

Steganography protects the data by concealing it in an image, audio, or video [27,28,38].

The way forward

First and foremost, all authors, researchers, and editors should be aware of the strengths as well as the weaknesses of AI tools [23]. Blanket banning will not prove to be a good solution as it will encourage the undisclosed use, thus compromising transparency and discouraging researchers not proficient in English who are getting legitimate help from these LLM tools. Detecting text generated by these LLM models is extremely difficult and will rapidly overcome even the waterproofing methods [23,25,27,31,32,48]. Disclosure of the use of LLM and chatbots should be done in the material and methods section, citation, and reference by mentioning and attaching relevant assistance as supplementary material [25,31]. A multipronged approach under the guidance of international agencies is suggested for framing robust ethical regulations and norms, developing rigorous review and assessment mechanisms, and training and enhancing the skills of researchers [26]. The future of scholarly publishing will be AI-dependent as it has the potential to transform the entire research process [23,39,43,44]. One of the solutions for mis-handled images is to make the authors submit raw images, or the use of AI-based tools to check image authenticity [26,35]. As far as image integrity is concerned, many publishing houses are using AI tools, namely, imageTwin, ImaCheck, and Proofis, in routine checks before publishing [35].

Once Google revolutionized access to medical literature and enabled faster, more informed research, AI-driven chatbots can serve as the next leap forward in information retrieval. Knowledge synthesis, hypothesis generation, assistance with study design and statistics, and writing and language support should be done with caution, wisdom, and transparency.

A SWOT Analysis was done to provide a comprehensive overview of ethical considerations in publication using AI (Table 1). Strategies should be devised by regulating agencies to address the weaknesses and threats in the form of robust ethical regulations and norms, developing rigorous review and assessment mechanisms, and training and enhancing the skills of researchers.

**Table 1:** SWOT analysis of ethical consideration in publication using AI. AI: artificial intelligence

SWOT component	Details	Score (1–5)
Strengths	Identify research ideas. Refine the abstract/manuscript. Analyze vast complex data. Develop an algorithm for peer review	4
Weaknesses	Limited accuracy with unreliable output. Plagiarism, falsification, and fabrication risk. Lack of critical human judgment. Data privacy concerns	4
Opportunities	Summarize vast medical literature. Good assistant for experienced researchers. Help non-native English speakers. Develop AI tools to detect research misconduct	5
Threats	Ethical concerns with the legal identity of AI. Lack of transparency/regulatory compliance. Risk of eroding trust in publishing. Need for course correction in publishing	5

## Conclusions

The scoring of the SWOT analysis reflects the current trends and perceived impact of AI in research, with the opportunities and threats scoring being higher due to their broad implications on the future of ethical publishing of scientific literature. The use of AI should be limited only to refining and editing the text, and the generated text should be cross-checked for facts and rewritten in one’s own language. While using these tools, the due rights of authors and publishing houses of copyright, trademark, and other rights should be respected. The information about the AI-generated material in manuscripts needs to be disclosed to the publisher and readers. The regulatory agencies should ensure that there is emphasis on the importance of human critical thinking in academic publications, and our autonomy as human academics should not be challenged. Above all, humans should remain accountable for scientific work. Let us not make this AI vs. humans, but AI collaborating with humans for the creation of more valuable scientific literature.
